# Immunization of Mice with Recombinant Protein CobB or AsnC Confers Protection against *Brucella abortus* Infection

**DOI:** 10.1371/journal.pone.0029552

**Published:** 2012-02-24

**Authors:** Simei Fu, Jie Xu, Xianbo Li, Yongfei Xie, Yefeng Qiu, Xinying Du, Shuang Yu, Yaoxia Bai, Yanfen Chen, Tongkun Wang, Zhoujia Wang, Yaqing Yu, Guangneng Peng, Kehe Huang, Liuyu Huang, Yufei Wang, Zeliang Chen

**Affiliations:** 1 Department of Infectious Disease Control, Institute of Disease Control and Prevention, Academy of Military Medical Science, Beijing, China; 2 School of Public Health, Key Laboratory of Zoonosis, Ministry of Education, Institute of Zoonosis, Jilin University, Changchun, China; 3 Experimental Animal Center, Academy of Military Medical Science, Beijing, China; 4 College of Veterinary Medicine, Sichuan Agricultural University, Ya'an, China; 5 College of Veterinary Medicine, Nanjing Agricultural University, Nanjing, China; University of Louisville, United States of America

## Abstract

Due to drawbacks of live attenuated vaccines, much more attention has been focused on screening of *Brucella* protective antigens as subunit vaccine candidates. *Brucella* is a facultative intracellular bacterium and cell mediated immunity plays essential roles for protection against *Brucella* infection. Identification of *Brucella* antigens that present T-cell epitopes to the host could enable development of such vaccines. In this study, 45 proven or putative pathogenesis-associated factors of *Brucella* were selected according to currently available data. After expressed and purified, 35 proteins were qualified for analysis of their abilities to stimulate T-cell responses *in vitro*. Then, an *in vitro* gamma interferon (IFN-γ) assay was used to identify potential T-cell antigens from *B. abortus*. In total, 7 individual proteins that stimulated strong IFN-γ responses in splenocytes from mice immunized with *B. abortus* live vaccine S19 were identified. The protective efficiencies of these 7 recombinant proteins were further evaluated. Mice given BAB1_1316 (CobB) or BAB1_1688 (AsnC) plus adjuvant could provide protection against virulent *B. abortus* infection, similarly with the known protective antigen Cu-Zn SOD and the license vaccine S19. In addition, CobB and AsnC could induce strong antibodies responses in BALB/c mice. Altogether, the present study showed that CobB or AsnC protein could be useful antigen candidates for the development of subunit vaccines against brucellosis with adequate immunogenicity and protection efficacy.

## Introduction

*Brucella* species are responsible for brucellosis, a worldwide zoonotic disease causing abortion in domestic animals (sheep, cattle, and goats) and Malta fever in humans [1], [2]. Humans are usually infected by *Brucella* through contact with infected animals or their products. Therefore, prevention of human brucellosis depends predominantly on the control of the disease in animals. Test and slaughter programs in conjunction with vaccination are the most important methods for control of animal brucellosis. Currently, *B. abortus* S19 or *B. abortus* RB51 is used to immunize cattle, whereas the *B. melitensis* Rev.1 strain is used to immunize goats and sheep in many countries. In general, the use of live attenuated organisms as vaccines possesses some problems in terms of safety, e.g., the potential of the organism to revert to its original virulent forms and the shedding of the organism into the environment. Particularly, the smooth vaccine strains induce antibodies responses that interferes the serological diagnosis in the test and slaughter programs. Some efforts are being made to overcome these problems by targeted deletion of antigenic proteins required for survival, with the aim to alleviate virulence and provide targets for differential diagnosis of infection and immunization [3], [4], [5].

Subunit vaccines have several advantages over live attenuated vaccines: they are completely inert, definite compositions, controllable productions and highly homogeneity. In addition, the subunit vaccines, by inducing an immune response to a single protein, would make it possible for the development of diagnostic tests that could differentiate vaccinated animals from infected animals. Subunit vaccines have been the *Brucella* research focus for many years. Several protein antigens are shown to induce protective immune response in mice model. The recent examples include the outer membrane protein combination of Omp16 and Omp19 for protection against *B. abortus* infection [6], and Omp28 with CpG oligonucleotides against *B. abortus* challenges [7]. Many of these studies are focused on the limited protein antigens. For development of subunit vaccines, screening and evaluation of protective antigens are the most important fundamental tasks. While many proteins have been assessed as potential protective antigens against *Brucella* infection, only a few have been shown to induce significant protection [8], [9]. The degree of protection efficiencies depends on the ability of the candidate antigens to direct immunity towards a Th1-type response [10], [11], [12], [13], [14], [15]. Therefore, proteins that induce T-cell meditated immune responses should be candidates for such vaccines. Identification and characterization of this type of antigens will greatly promote discovery of subunit vaccine candidates.

As an intracellular bacterium, *Brucella* has some unique characteristics. There are no any plasmids and toxins in *Brucella* that play important roles in interaction between bacteria and hosts. However, *Brucella* has great capabilities to survive in multiple types of host cells [16]. The proteins that involved in intracellular survival are important virulence factors of *Brucella*. For many pathogenic bacteria, antigenic virulence proteins are usually protective antigens. Actually, there are several proteins that provide protection against infections also play important roles in *Brucella* virulence. Therefore, virulence proteins that induce T-cell mediated immune responses might be protective antigen candidates. To identify protective antigens, in the present study, proven or putative virulence related proteins of *Brucella* were selected for antigenicity analysis. These proteins were expressed in *E. coli* and purified, and then used to stimulate splenocyte from *B. abortus* live vaccine S19 immunized mice. The proteins that greatly induce secretion of IFN-γ were used to immunize BALB/c mice and tested for its capability to provide protection against *B. abortus* 544 infection.

## Materials and Methods

### Mice and Ethics Statement

Female 6-week-old BALB/c mice obtained from The Animal Center of Military Medical Sciences. All animals were handled in strict accordance with Experimental Animal Regulation Ordinances defined by China National Science and Technology Commission, and the animal work was approved by Beijing Institute of Disease Control and Prevention animal ethics committee (Ethical Approval BIDCP003-2011). Animals are provided with humane care and healthful conditions during their stay in the facility. All individuals who use animals receive instruction in experimental methods and in the care, maintenance and handling of mice, and are under the committee's supervision.

### Bacterial strains and plasmids

*B. abortus* 544 and S19 were cultured in Tryptic Soy Broth (TSB) or Tryptic Soy Agar (TSA). *E. coli* strain ER2566 was grown on Luria–Bertani (LB) medium. Plasmid pDONR201 (Invitrogen) was used for Gateway® BP Reaction entry clones construction and pHXGWA for Gateway® LR Reaction expression clones [17].

### Cloning, expression, and purification of His-tagged recombinant proteins

45 proven or putative pathogenesis-associated proteins according to currently available data [18], [19], [20], [21], [22], [23], [24], [25] and a known protective antigen Cu-Zn SOD (BAB2_0535) [26] were selected to be expressed in *E. coli* ER2566. Briefly, the selected ORFs were amplified by PCR from genomic DNA of *B. abortus* 544 with specific primers tailed with adaptors (Table S1). Then, the products were amplified with adaptor primer AttB1 (5′-GGG GAC AAG TTT GTA CAA AAA AGC AGG CT-3′) and AttB2 (5′-GGG GAC CAC TTT GTA CAA GAA AGC TGG GT-3′) to attach the recombination sequences. The amplified DNA fragments were cloned into the pDONR201 vector by using Gateway cloning technology. The sequences of the cloned ORFs were verified by DNA sequencing. Then, the ORFs were sub-cloned into expression plasmid pHXGWA and the recombinant proteins were expressed in *E. coli* ER2566 as N-terminally His-tagged fusion proteins. The expression of the recombinant proteins was verified by SDS-PAGE. The recombinant fusion proteins were then purified by affinity chromatography on Ni^2+^-conjugated chelating Sepharose. Proteins expressed as inclusion bodies were solubilized with 6 M urea and refolded by serial dialysis against 4, 2, 1 and 0 M of urea in PBS. The purified proteins were finally solubilized in PBS-1% glycine (0.01 M; pH 8.0). The purity of the purified proteins was assessed by SDS-PAGE and the concentration was quantified.

### Mice immunization, splenocyte stimulation and IFN-γ secretion detection

*B. abortus* live attenuated vaccine S19 was cultured in TSB at 37°C to logarithmic phase, cells were precipitated by centrifugation and then washed with PBS twice. Groups of six female 6-week-old BALB/c mice were immunized intraperitoneally (i.p.) with 200 µl bacterial suspension containing 5×10^6^ CFU of S19 per mouse or 200 µl of PBS as a control. Twenty-eight days later, the sera were collected for determining the IgG titers of antibody against heat-killed and sonicated S19 whole-cell antigen by enzyme-linked immunosorbent assay (ELISA). On the 35th day, the immunized mice and nonimmunized control mice were killed humanely and their spleens were aseptically recovered. Single cell suspensions of mixed splenocytes from 3 immunized mice with higher antibodies titers or 3 control mice were obtained by homogenization. Cells were pelleted at 1000 rpm for 10 min, and then 10 ml red blood cell lysis buffer (150 mM NH_4_Cl, 1 mM KHCO_3_, 0.1 mM Na_2_EDTA [pH 7.3]) was added to the pellet for 5 min. After washing three times with PBS, the cells were resuspended in complete RPMI 1640 medium (GIBCO BRL) supplemented with 10% (v/v) heat-inactivated fetal bovine serum and 2 mM L-glutamine. The cells were cultured in 96-well plates for 72 h at a concentration of 5×10^5^ cells/well in the presence of 5 µg of purified recombinant proteins, 0.5 µg of concanavalin A (ConA, positive control) or medium alone (negative control), respectively. Then the plates were centrifuged at 1000 rpm for 10 min, the clear culture supernatants were collected and stored at −20°C. IFN-γ was determined by using an ELISA Quantikine Mouse kit (R&D Systems). All assays were performed in triplicate and the concentration for IFN-γ in the culture supernatants was calculated by using a linear regression equation obtained from the absorbance values of the standards according to the manufacturer's procedures.

### Immunization and protection experiments

Female 6-week-old BALB/c mice were immunized by the intraperitoneal route with purified recombinant proteins. Briefly, mice were immunized with 200 µl of recombinant proteins (30 µg) or PBS (negative control) mixed with complete Freund's Adjuvants (CFA, Sigma) on day 0 and with incomplete Freund's Adjuvants (IFA, Sigma) on day 15. The positive control group was immunized on day 15 with 1×10^5^ CFU of *B. abortus* S19 mixed with IFA. 30 days after the last immunization, mice were challenged i.p. with 2×10^4^ CFU of *B. abortus* virulent strain 544. 30 days post the challenge, mice were killed by cervical dislocation and spleens were removed aseptically and homogenized with PBS containing 0.1% Triton X-100. The homogenates were serially diluted and plated on TSA, and the CFU were counted after 5 days of incubation at 37°C.

### Humoral immune responses

Sera for antibody response detection were obtained at 0, 15, 30 and 45 days after the first immunization. Serum reaction against purified recombinant proteins were determined by indirect ELISA as described previously [8], [9].

### Statistical analysis

Cytokine production in vitro was expressed as mean cytokine concentration ± SD. The protective efficiency was expressed as the mean log_10_ CFU ± SD. The differences between groups were analyzed by ANOVA followed by Tukey's honestly significant difference post test comparing all groups to one another. For ANOVA, *P* values of <0.05 were considered statistically significant.

## Results

### Cloning, expression and purification of virulence-associated proteins of *Brucella*

**Figure 1:**
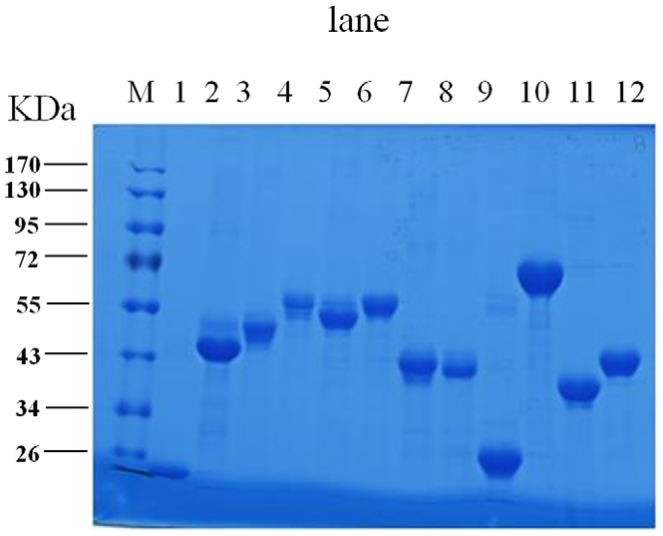
SDS-PAGE of the recombinant proteins. The expression of recombinant fusion proteins were analyzed by SDS-PAGE. M, molecular size protein markers; lane 1–12, the purified fusion proteins: BAB1_0063, BAB1_0116, BAB1_0381, BAB1_0512, BAB1_0553, BAB1_0560, BAB1_0597, BAB1_0722, BAB1_0812, BAB1_0917, BAB1_1108, BAB1_1124.

According to currently available data, 45 proven or putative pathogenesis-associated factors were selected (Table S1). In addition, a known protective antigen Cu-Zn SOD (BAB2_0535) was also cloned and expressed. The selected 46 ORFs were amplified by PCR and cloned into the pDONR201 vector by using Gateway cloning technology. The sequences of the cloned ORFs were verified by DNA sequencing. Then, the amplified DNA sequences of the ORFs for these proteins were cloned into pHXGWA to generate expression plasmids that were then transformed into *E. coli* ER2566 to express the recombinant fusion proteins with His tags. According to the SDS-PAGE analysis, 35 ORFs were successfully expressed, with 19 (54.3%) of them being expressed as inclusion bodies (data not shown). All the proteins were purified by Ni^2+^-conjugated chelating chromatography. Proteins expressed as inclusion bodies were solubilized with urea and refolded by dilution after being purified. All purified proteins were finally solubilized in PBS–1% glycine. The purity of the purified proteins was assessed by SDS-PAGE (Figure 1) and the concentration was quantified (data not shown). In the end, 35 proteins were successfully purified and qualified for subsequent analysis of their abilities to stimulate T-cell responses (Table S1).

### Proteins that induce IFN-γ secretions in splenocytes of S19 immunized mice

**Figure 2:**
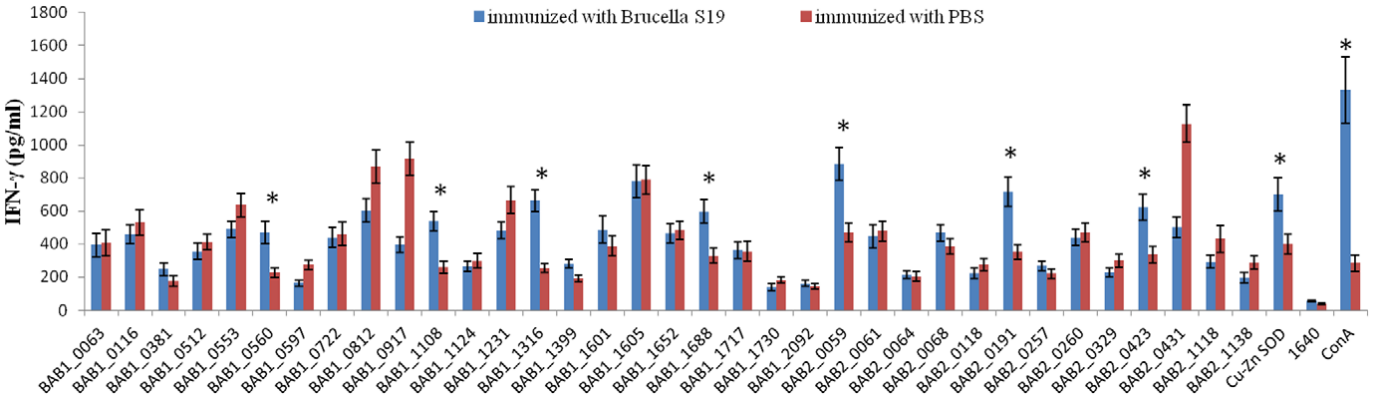
IFN-γ secretion by splenocytes stimulated with the recombinant *B. abortus* proteins. Spleen cells from mice immunized with S19 or PBS were stimulated in vitro with recombinant proteins, ConA (positive control), or complete medium (negative control). Supernatants were collected and IFN-γ was determined by using an ELISA Quantikine Mouse kit (R&D Systems). All assays were performed in triplicate and the concentrations for IFN-γ in the culture supernatants were calculated by using the quantification formula. Significant differences between S19-immunized mice and PBS-immunized mice are indicated as follows: *, P<0.001.

To assess the T-cell immunity inducing activity of the 35 proteins, sensitized splenocytes were firstly prepared. BALB/c mice were i.p. immunized with live vaccine strain S19. On the 28th day post the immunization, sera from immunized mice were collected and the IgG levels were measured by ELISA. The results showed the IgG titer reached at least 10000 (data not shown), suggesting that the single injection induced immune responses in mice. 35 days post the immunization, spleens were removed and cells were isolated. The splenocytes were stimulated with each of the purified recombinant proteins, complete medium (negative control) or ConA (positive control), and secretion of IFN-γ was assayed. As shown in Figure 2, compared with cells from control group immunized with PBS, splenocytes from S19-immunized mice secreted significant higher levels of IFN-γ (P<0.001) when stimulated by BAB1_0560, BAB1_1108, BAB1_1316, BAB1_1688, BAB2_0059, BAB2_0191, and BAB2_0423, similarly with the known protective antigen Cu-Zn SOD. The results indicated that these 7 proteins were good candidates for further protection analysis (Table 1).

**Table 1:** Proteins that induced strong T-cell responses in mice immunized with S19.

Locus	Protein name	The location of Protein	Description
BAB1_0560	-	Cytoplasm	phosphomannomutase
BAB1_1108	-	Unknown	predicted acyl-CoA transferase
BAB1_1316	CobB	Cytoplasm	cobyrinic acid a,c-diamide synthase
BAB1_1688	AsnC	Unknown	transcriptional regulatory protein, AsnC family
BAB2_0059	VirB10	Unknown	channel protein virB10 homolog
BAB2_0191	-	Cytoplasm	HAD superfamily protein involved in N-acetyl-glucosamine catabolism
BAB2_0423	GntR	Cytoplasm	transcriptional regulator, gntR family

### CobB and AsnC induce protective immune responses against *B. abortus* 544 challenge

Then, the 7 proteins were used to immunize BALB/c mice and their protection efficiencies were evaluated. At 45 days after the first immunization, the vaccinated mice were challenged with virulent strain *B. abortus* 544, and 30 days post the challenge, *Brucella* in spleen were isolated and enumerated. Protection efficiency was defined as the difference between the numbers of viable bacteria recovered from spleens of immunized mice and those recovered from spleens of mice receiving PBS. Mice immunized with BAB1_1316 (CobB) or BAB1_1688 (AsnC) plus adjuvant exhibited a significant degree of protection (P<0.001) when compared with controls receiving PBS, with 1.16 and 1.89 units of protection, respectively (Table 2). As expected, the known protective antigen Cu-Zn SOD and the license vaccine S19 offer significant protection (1.30 and 1.02 units, respectively). The remaining 5 recombinant proteins could not provide significant protection against virulent *B. abortus* challenge (Table 2).

**Table 2:** Protection against *B. abortus* 544 infection induced by recombinant *B. abortus* proteins immunization.

Vaccine (n = 5)	Adjuvant	log_10_ *B. abortus* at spleen	Units of protection^a^
BAB1_1316	CFA/IFA	5.71±0.32	1.16*
BAB1_1688	CFA/IFA	4.98±0.72	1.89*
BAB1_0560	CFA/IFA	6.68±0.30	—
BAB1_1108	CFA/IFA	6.48±0.21	—
BAB2_0059	CFA/IFA	6.26±0.61	—
BAB2_0423	CFA/IFA	6.58±0.14	—
BAB2_0191	CFA/IFA	6.52±0.70	—
Cu-Zn SOD	CFA/IFA	5.57±0.28	1.30*
S19	IFA	5.85±0.85	1.02*
PBS	CFA/IFA	6.87±0.24	0

a Units of protection were obtained by subtracting the mean log_10_CFU/spleen of the vaccinated group from the mean log_10_CFU/spleen of the control (PBS) immunized group.

* p value<0.05.

### CobB and AsnC induce antibodies responses

**Figure 3:**
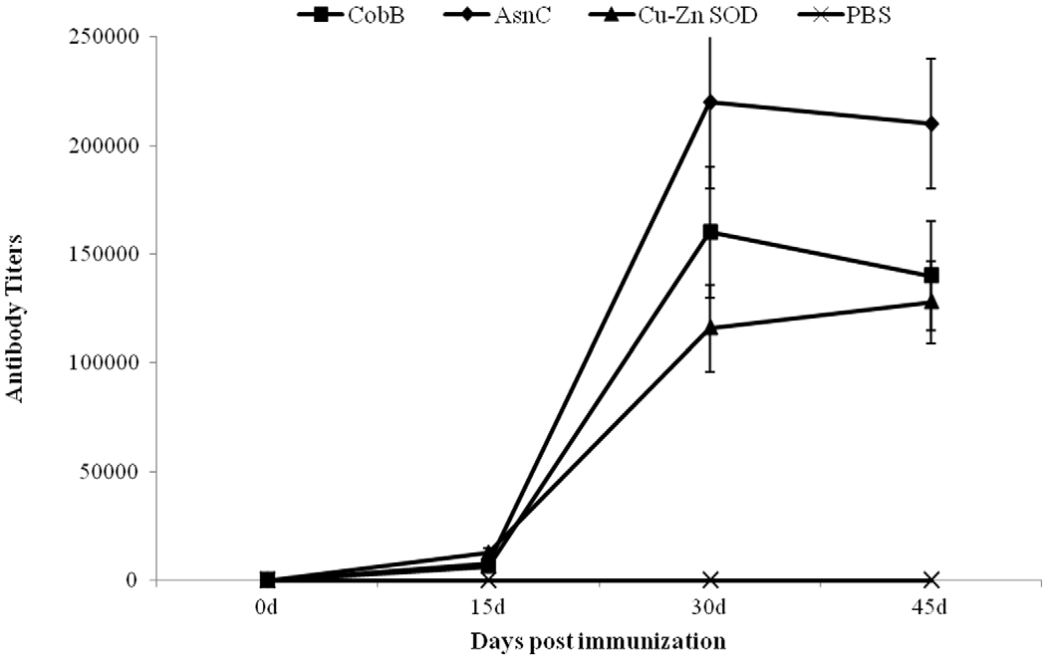
Humoral immune response induced by CobB, AsnC, and Cu-Zn SOD. BALB/c mice (n = 5) were inoculated intraperitoneally with purified recombinant proteins plus adjuvant. At 0, 15, 30, and 45 days post the immunization, serum samples were collected and IgG antibody titers were determined by ELISA. The values are mean titers ± SD of antibodies from the five mice.

To further evaluate the immunogenic characteristics of CobB and AsnC, the production of antigen-specific antibodies was measured in the sera of the immunized mice by indirect ELISA. For comparison, the known protective antigen Cu-Zn SOD was also included. As shown in Figure 3, immunization with CobB or AsnC plus adjuvant elicited a humoral immune response that was detectable 15 days (IgG mean titer: 6400 and 8000, respectively) after the first immunization and reached a maximum at day 30 post the vaccination (IgG mean titer: 160000 and 220000, respectively), higher than the positive control protein Cu-Zn SOD. The results indicated that immunization with CobB or AsnC plus adjuvant could also induce humoral immune response.

## Discussion

*Brucella* is a genus of intracellular pathogens that preferentially infect macrophages. Successful elimination of intracellular pathogens relies on capability of vaccines to generate potent cell mediated immunity. In particular, Th1 immune responses characterized by production of gamma interferon (IFN-γ) are associated with protective immunity to *Brucella* [27], [28]. Thus identification and characterization of *Brucella* antigens which present T-cell epitopes to the host will contribute greatly to development of subunit vaccines against *Brucella* infection.

The availability of complete bacterial genome sequences makes a push for reverse vaccinology to be put into practice [29], [30]. Immunoproteomics and protein microarrays were used to identify novel immunogens that induced humoral immune responses in a high-throughput manner [9], [31], [32], [33], [34]. However, the approach to identify T-cell antigens on a large scale is just being underway [9], [35]. In this study, proteins associated with *Brucella* pathogenesis were expressed in *E. coli*. Then, their abilities to induce T-cell responses were tested. For the proteins that could stimulate strong IFN-γ responses, immunogenicity and protective ability were further evaluated. This reverse vaccine methodology is ideal for the rapid identification of T-cell antigens and development of subunit vaccines for brucellosis.

In this study, 45 proven or putative pathogenesis-associated proteins were selected for in vitro expression. Previous studies demonstrated that vaccination of mice with *E. coli* expressing *Brucella* Cu-Zn SOD was able to induce antigen specific Th1 immune responses and conferred a significant level of protection against virulent *B. abortus* challenge [26]. So Cu-Zn SOD was also expressed and used as positive experimental control. In the end, 35 of 45 proteins were successfully expressed and purified. Among these 35 proteins, 7 proteins could stimulate stronger IFN-γ responses in mice immunized with S19, indicating their abilities to induce T-cell responses. The reasons for the other 28 proteins not recognized as T-cell immunogens in this study include several possibilities. First, these proteins could not induce T-cell responses in deed. Second, in our study, the expressed proteins were recombinant and had lost their native conformations that might be important for T-cell response induction. Third, in the in vitro T-cell stimulation assays, the same concentration of each protein was used. But the optimal concentration for in vitro stimulation may be different for these proteins. Thus our study may ignore some proteins which also induce T-cell responses.

Very interestingly, we found that some proteins induced greatly higher levels of IFN-γ in splenocytes of PBS immunized mice than those of S19 immunized ones. For example, BAB2_0431 and BAB1_0917 induced 2.26 and 2.31 folds higher IFN-γ in PBS immunized mice than that in S19 immunized ones. This was not observed in our previous studies on *Yersinia pestis*, where all tested proteins induced higher or identical levels of IFN-γ in vaccine strain immunized mice than control mice [36]. We thought there might be two possibilities: interference of host immune response and acquired immune tolerance. *Brucella* is an intracellular bacterium that could cope with host immune response for its survival. A Toll/Interleukin-1 receptor (TIR) domain containing protein mimics TcpB has been shown to inhibit TLR2 and TLR4 mediated NF-kB activation. In vivo mouse studies indicated that *tcpB* mutant was defective in systemic spread in the early stages of infection [37]. It is possible that some of these proteins interfere with the secretion of IFN-γ. Acquired immune tolerance might be another reason, which is characterized by a specific non-reactivity to a given antigen that would likely induce cellular or humoral immunity in other circumstances. It will be very interesting to probe into the roles of these proteins in immune response regulation or immune tolerance.

The protective efficiencies of 7 recombinant proteins that could induce T-cell responses were evaluated in mice model. At 4 weeks post challenge, mice immunization with BAB1_1316 (CobB) or BAB1_1688 (AsnC) plus adjuvant demonstrated 1.16 and 1.89 units of protection (P<0.001) compared with PBS controls. However, different from previously studies, license vaccine S19 only showed 1.02 units of protection [6], [8]. This difference may be attributed to the distinction in the status of mice and challenging strain 544. In the present study, we found that the bacterial load in the PBS group is at least 1 log higher than that observed in other studies, implying the possibility of increased virulence of strain 544. On the other hand, the spontaneous mutation of some genetic factors in mice might also affect the susceptibility of the mice to intracellular pathogens. For example, mutations in Nramp1 gene influence innate resistance to *Mycobacterium* species [38]. Spontaneous mutations in these genes might affect the sensitivity of mice to the challenging strain 544. Therefore, both the genetic background alterations of the mice and/or the virulence of challenging strain 544 could affect the bacterial load in the spleen.

In the present study, the known protective antigen Cu-Zn SOD was also used as positive control, using the same immunization programs with candidate proteins. Results showed that Cu-Zn SOD conferred a similar degree of protection with CobB, being higher than that of S19. In general, acellular vaccines such as purified recombinant proteins and synthetic peptides are usually unable to confer a higher degree of protection than live attenuated vaccines [39]. The reduced effectiveness of the acellular vaccines might be related to inadequate processing and presentation of the antigen. However, live attenuated vaccine strains could infect the host cells efficiently and produce endogenous antigens in antigen-presenting cells. Right adjuvant and adequate delivery systems can augment cell-mediated immune responses to the target antigen and enhance the protection efficiency of subunit vaccines. Although in most cases, live attenuated vaccines confer better protection against *Brucella* infection than subunit vaccines. Actually, *Brucella* subunit vaccines that have similar or higher protection efficiency than live attenuated vaccine strains were also observed in recent reports [40], [41]. The reasons for the higher protection efficiency of subunit vaccines might include the high concentration of immunization antigens, the repeated immunization and the use of adjuvant or delivery system that induce great protective immune responses.

BAB1_1316 (CobB) is a cobyrinic acid a, c-diamide synthase, related to vitamin B12 synthesis, and BAB1_1688 (AsnC) is a member of the transcriptional regulatory proteins, both of which have recently been demonstrated to be involved in the pathogenesis of *Brucella* [18]. *Brucella* mutant deficient for either of the two proteins could not establish infection at early infection stages, indicating that the two proteins are important for *Brucella* early stage of infection. From this point of view, it could be putatively predicted that virulence proteins that play essential roles at early infection stages are more possibly protective antigens for *Brucella*. To further evaluate the vaccine potential of recombinant proteins CobB and AsnC, the antibodies response was also investigated. Results showed that immunization with CobB or AsnC plus adjuvant could induce remarkable titers of total IgG, higher than the positive control protein Cu-Zn SOD. Thus immunization of CobB or AsnC plus adjuvant could induce both humoral and cellular immune responses.

To further confirm the vaccine candidate property of the two proteins, the sequences of them were compared among different *Brucella* species. A BLAST analysis revealed that CobB and AsnC were highly conserved among different *Brucella* species in term of both nucleotide acid and amino acid (data not shown). The results indicated that CobB and AsnC would be suitable candidates for developing vaccines against different *Brucella* infections.

In conclusion, our results indicate that pathogenesis-associated proteins CobB and AsnC could be potential protective antigens for the development of subunit vaccines against brucellosis since they could elicit both humoral and cellular immune responses, and confer protection against virulent *B. abortus* challenge. Further studies will be focus on develop efficient adjuvant or antigen delivery systems to further evaluate the protective immunity of CobB and AsnC.

## Supporting Information

Table S1 Proteins information and amplification primers of the selected proteins
(DOC)
