# Supplementary material for: Immunization of Mice with Recombinant Protein CobB or AsnC Confers Protection against Brucella abortus Infection
Source: PLoS One. 2012 Feb 24;7(2):e29552. doi: 10.1371/journal.pone.0029552 (PMC3286461; doi:10.1371/journal.pone.0029552)
Supplement: Table S1 — Proteins information and amplification primers of the selected proteins (DOC) [file pone.0029552.s001.doc]

Supplementary Table S1.

Proteins information and amplification primers of the selected proteins

| **Sequence Definition ^#^** | **Function** | **Sense Primer** | **Anti-sense Primer** | **Length**  **(bp)** | **Reference** |
| --- | --- | --- | --- | --- | --- |
| BAB1_0063 | Hypothetical Cytosolic Protein | GTACAAAAAAGCAGGCTTAATGGCATTCGAGGACATCAAG | TGTACAAGAAAGCTGGGTCGGAATCGAAATCTTGTTCGATGC | 186 | 19 |
| BAB1_0116 | Omp25C | GTACAAAAAAGCAGGCTTAGACGCCGTCATTGAACAGG | TGTACAAGAAAGCTGGGTCGAACTTGTAAGCGACACCG | 615 | 23 |
| BAB1_0381 | transcriptional regulator, lysR family | GTACAAAAAAGCAGGCTTAAAGCTTTGCGCTTCTGG | TGTACAAGAAAGCTGGGTCCGCCTCGCGGATCAGC | 969 | 20 |
| BAB1_0512 | GMP phosphodiesterase A-related protein | GTACAAAAAAGCAGGCTTACTGGTCGGAAAAGGACTCC | TGTACAAGAAAGCTGGGTCTTGCTTCTTCTTCACTGTAG | 1,506 | 19 |
| BAB1_0553 | mannosyltransferase | GTACAAAAAAGCAGGCTTAATGCGAATTGGTGTCGACG | TGTACAAGAAAGCTGGGTCATAGGTCATGAGCTTAGATTCTCTG | 1,116 | 18 |
| BAB1_0560 | phosphomannomutase | GTACAAAAAAGCAGGCTTATTGAGGAAAACCACAGAG | TGTACAAGAAAGCTGGGTCAACCGTATCGCCATTCGC | 1,305 | 19 |
| BAB1_0597 | Hypothetical Cytosolic Protein | GTACAAAAAAGCAGGCTTAATGACGAGGCAGATTTCGG | TGTACAAGAAAGCTGGGTCGGGCGATAATCCTCCGC | 771 | 19 |
| BAB1_0629 * | sensor protein phoQ | GTACAAAAAAGCAGGCTTACTGGTCGTGGTCGCCACT | TGTACAAGAAAGCTGGGTCATCTTGCGTCAACGGCAAC | 1,304 | 19 |
| BAB1_0722 | Omp25 | GTACAAAAAAGCAGGCTTAGCCGACGCCATCCAGGAACAG | TGTACAAGAAAGCTGGGTCGAACTTGTAGCCGATGCCG | 570 | 23 |
| BAB1_0812 | hypothetical protein | GTACAAAAAAGCAGGCTTAATGACAATCGAAGATCTGCAG | TGTACAAGAAAGCTGGGTCTCGCGGTGGAATCGAATAG | 402 | 19 |
| BAB1_0917 | trigger factor | GTACAAAAAAGCAGGCTTAACCGAAACGCTCAATGAAGG | TGTACAAGAAAGCTGGGTCAGCCTCTTCGGACTTGCC | 1,422 | 19 |
| BAB1_1058 * | VceC | GTACAAAAAAGCAGGCTTAGATGCCGCCGCGGTAG | TGTACAAGAAAGCTGGGTCTTCTCCCTTGGTCAACTCAC | 1,068 | 22 |
| BAB1_1108 | predicted acyl-CoA transferase | GTACAAAAAAGCAGGCTTACAATTTGCGGGCGGGCTTTC | TGTACAAGAAAGCTGGGTCAGCGCCGTGTTCATTTAG | 1,161 | 19 |
| BAB1_1124 | transcriptional regulator, gntR family | GTACAAAAAAGCAGGCTTAAAGGCAAAGACCAGACG | TGTACAAGAAAGCTGGGTCTACAAGATCATGGATGGG | 756 | 20 |
| BAB1_1231 | DNA-directed RNA polymerase alpha subunit | GTACAAAAAAGCAGGCTTAATCCAGAAGAACTGGCAGG | TGTACAAGAAAGCTGGGTCATATTGGTCTTCGTAGCGC | 1,008 | 19 |
| BAB1_1280 * | hypothetical protein | GTACAAAAAAGCAGGCTTAATGCCGGGTGCAATAAACC | TGTACAAGAAAGCTGGGTCGAGATCGACGTCGAGAATGG | 252 | 21 |
| BAB1_1316 | cobyrinic acid a,c-diamide synthase | GTACAAAAAAGCAGGCTTAATGAAGGGATTCATGATTGCCG | TGTACAAGAAAGCTGGGTCCGCCGCTTCTCCGCTAA | 1,308 | 19 |
| BAB1_1399 | ketol-acid reductoisomerase | GTACAAAAAAGCAGGCTTAATGCGCGTTTATTACGATCGG | TGTACAAGAAAGCTGGGTCGTTGCGGGCTTTATCGACC | 1,017 | 19 |
| BAB1_1601 | Periplasmic dipeptide transport protein | GTACAAAAAAGCAGGCTTAATGGGGTGCGCGCGG | TGTACAAGAAAGCTGGGTCCATCGTCGGCGGGATCGG | 1,056 | 19 |
| BAB1_1605 | nodulation protein nolR | GTACAAAAAAGCAGGCTTAACTAACAAAGTTACTTTTTAT | TGTACAAGAAAGCTGGGTCCACCGCCATCATTTCAC | 333 | 20 |
| BAB1_1652 | VceA | GTACAAAAAAGCAGGCTTAACCCAATGCGATGCAAAGC | TGTACAAGAAAGCTGGGTCGTGGCCCTTGTCCTTG | 237 | 22 |
| BAB1_1688 | transcriptional regulatory protein, asnC family | GTACAAAAAAGCAGGCTTATCCGCTTTAACTTGGGAC | TGTACAAGAAAGCTGGGTCCAGCGGGACGGCTGTG | 489 | 19 |
| BAB1_1717 | hypothetical protein | GTACAAAAAAGCAGGCTTAATTAGCGAACAGGGTCAGATG | TGTACAAGAAAGCTGGGTCTTGTGTGCCGAAAGCG | 522 | 19 |
| BAB1_1718 * | OPGC | GTACAAAAAAGCAGGCTTAATGGCGATTGAAACTGCAAA | TGTACAAGAAAGCTGGGTCGTATCGCTGCATTGCTGG | 1,200 | 19 |
| BAB1_1730 | transcriptional regulator, gntR family | GTACAAAAAAGCAGGCTTAATGAATCAGAATGTCCCAGCCT | TGTACAAGAAAGCTGGGTCCCCCACAATGGCGAAGGAA | 690 | 20 |
| BAB1_1731 * | bioY protein | GTACAAAAAAGCAGGCTTAATGGCGACCTTCGCTCCG | TGTACAAGAAAGCTGGGTCCGCCTTGCCGTCGCG | 573 | 21 |
| BAB1_1894 * | transcriptional regulator, gntR family | GTACAAAAAAGCAGGCTTATTGGAAGCTGTCGAAGC | TGTACAAGAAAGCTGGGTCAGGGTCTGCGGACTGG | 1,407 | 20 |
| BAB1_1922 * | dihydrolipoamide acetyltransferase | GTACAAAAAAGCAGGCTTAATGGCTACCGAAATTCGCG | TGTACAAGAAAGCTGGGTCGAGATCGAGAACCAGACGT | 1,224 | 21 |
| BAB1_2092 | transcriptional regulatory protein chvI | GTACAAAAAAGCAGGCTTAAAGGAAGCTTCGGCAACG | TGTACAAGAAAGCTGGGTCCGCTTCCCGGAAACGATAAC | 714 | 21 |
| BAB2_0059 | channel protein virB10 homolog | GTACAAAAAAGCAGGCTTACCGGATGATACGCCGGC | TGTACAAGAAAGCTGGGTCCTTCGGTTTGACATCATACACAC | 792 | 18 |
| BAB2_0061 | VirB8 | GTACAAAAAAGCAGGCTTATTTGGACGCAAACAATCTCC | TGTACAAGAAAGCTGGGTCTTGCACCACTCCCATTTCTG | 714 | 24,25 |
| BAB2_0064 | VirB5 | GTACAAAAAAGCAGGCTTAAAGAAGATAATTCTCAGCTTCG | TGTACAAGAAAGCTGGGTCATAGGCGGCTTCCAGTG | 711 | 24 |
| BAB2_0068 | attachment mediating protein virB1 homolog | GTACAAAAAAGCAGGCTTAGTGCCATTCCTTGTCCTCG | TGTACAAGAAAGCTGGGTCGAAAACAACTACGCCGTCC | 711 | 18 |
| BAB2_0118 | transcriptional activator, luxR family | GTACAAAAAAGCAGGCTTAGCGCTTCTAACCCGCATCC | TGTACAAGAAAGCTGGGTCGACGAGATGCTGTACCTCGG | 702 | 20 |
| BAB2_0172 * | pyruvate dehydrogenase complex repressor | GTACAAAAAAGCAGGCTTAATGGCCGACACAGTTT | TGTACAAGAAAGCTGGGTCTTCGCTCTCGTCTTCC | 810 | 19 |
| BAB2_0191 | HAD superfamily protein involved in  N-acetyl-glucosamine catabolism | GTACAAAAAAGCAGGCTTATTGAGGAAAATGATGATAC | TGTACAAGAAAGCTGGGTCATGTAGAGAAGCAATGGG | 849 | 19 |
| BAB2_0257 | 6-aminohexanoate-dimer hydrolase | GTACAAAAAAGCAGGCTTAATGAAGCGTCTATTCCGCTT | TGTACAAGAAAGCTGGGTCAAGCTTGTCTTCAAAATATTTCTGG | 768 | 19 |
| BAB2_0260 | cytochrome c oxidase polypeptide IV | GTACAAAAAAGCAGGCTTAATGGGTGGAACTGAAATGGC | TGTACAAGAAAGCTGGGTCCGAAAGAAGGAACCAGGCAA | 237 | 21 |
| BAB2_0329 | transcriptional regulator, lysR family | GTACAAAAAAGCAGGCTTAATGAAGCTGAGCAGGAGATTG | TGTACAAGAAAGCTGGGTCTTCCGGTTTGGAATGAACCAA | 930 | 20 |
| BAB2_0423 | transcriptional regulator, gntR family | GTACAAAAAAGCAGGCTTAATGGGAATTTGGATGAGAGGA | TGTACAAGAAAGCTGGGTCAGCAAAATAGTCCGGACGG | 786 | 20 |
| BAB2_0431 | D-galactarate dehydratase | GTACAAAAAAGCAGGCTTACATCTTCAGAATCTCGC | TGTACAAGAAAGCTGGGTCCACCGCACCGACCTG | 1,302 | 19 |
| BAB2_0535 | Superoxide Dismutase (Cu-Zn) | GTACAAAAAAGCAGGCTTAATGGTGCTTATGGCTTTTCCG | TGTACAAGAAAGCTGGGTCAAGCGGCTCAGGCTTATCG | 453 | 26 |
| BAB2_0584 * | sn-glycerol-3-phosphate transport system  ermease protein ugpA | GTACAAAAAAGCAGGCTTAATGCGGGAAGACGCATTC | TGTACAAGAAAGCTGGGTCTGAATAATGCACGCGCTTCT | 753 | 19 |
| BAB2_1098 * | flagellar hook protein | GTACAAAAAAGCAGGCTTAATGAGCCTCTACGGTATGATGC | TGTACAAGAAAGCTGGGTCTCTCTTCAGATTAACCAGCACGT | 1,188 | 21 |
| BAB2_1118 | homoprotocatechuate 2,3-dioxygenase | GTACAAAAAAGCAGGCTTAAGGAAAATCATGCCCTTGC | TGTACAAGAAAGCTGGGTCGATCGGCTGTGCCTTCAAC | 978 | 19 |
| BAB2_1138 | transcriptional regulator, gntR family | GTACAAAAAAGCAGGCTTAATGAATGTTGAATCGGATCATGC | TGTACAAGAAAGCTGGGTCCCTTGTCCGACGTGATAATCG | 732 | 20 |

# The sequence definition and their annotation were according to the *Brucella abortus* 2308 genome annotation.

* The proteins that were not expressed.
